# Escherichia coli Harboring *mcr-1* and *bla*_CTX-M_ on a Novel IncF Plasmid: First Report of *mcr-1* in the United States

**DOI:** 10.1128/AAC.01103-16

**Published:** 2016-06-20

**Authors:** Patrick McGann, Erik Snesrud, Rosslyn Maybank, Brendan Corey, Ana C. Ong, Robert Clifford, Mary Hinkle, Timothy Whitman, Emil Lesho, Kurt E. Schaecher

**Affiliations:** a Multidrug-resistant Organism Repository and Surveillance Network, Walter Reed Army Institute of Research, Silver Spring, Maryland, USA; b Department of Infectious Diseases, Walter Reed National Military Medical Center, Bethesda, Maryland, USA; c Department of Pathology, Walter Reed National Military Medical Center, Bethesda, Maryland, USA

## LETTER

The recent discovery of a plasmid-borne colistin resistance gene, *mcr-1*, in China heralds the emergence of truly pan-drug-resistant bacteria (1). The gene has been found primarily in Escherichia coli but has also been identified in other members of the Enterobacteriaceae in human, animal, food, and environmental samples on every continent (2–5). In response to this threat, starting in May 2016, all extended-spectrum-β-lactamase (ESBL)-producing E. coli clinical isolates submitted to the clinical microbiology laboratory at the Walter Reed National Military Medical Center (WRNMMC) have been tested for resistance to colistin by Etest. Here we report the presence of *mcr-1* in an E. coli strain cultured from a patient with a urinary tract infection (UTI) in the United States. The strain was resistant to colistin, but it remained susceptible to several other agents, including amikacin, piperacillin-tazobactam, all carbapenems, and nitrofurantoin (Table 1).

E. coli MRSN 388634 was cultured from the urine of a 49-year-old female who presented to a clinic in Pennsylvania on 26 April 2016 with symptoms indicative of a UTI. The isolate was forwarded to WRNMMC, where susceptibility testing indicated an ESBL phenotype (Table 1). The isolate was included in the first 6 ESBL-producing E. coli isolates selected for colistin susceptibility testing, and it was the only isolate to have a MIC of colistin of 4 μg/ml (all of the others had MICs of ≤0.25 μ/ml). The colistin MIC was confirmed by broth microdilution, and *mcr-1* was detected by real-time PCR (6). Whole-genome sequencing (WGS) of MRSN 388634 was performed using a PacBio RS II system and a MiSeq benchtop sequencer.

**TABLE 1:** Antibiotic resistance profile of MRSN 388634

Antibiotic(s)	MIC(s) (μg/ml)^*a*^
Amikacin	≤8, S
Amoxicillin/clavulanate	16/8, I
Ampicillin	>16, R
Aztreonam	>16, R
Cefazolin	>16, R
Cefepime	>16, R
Ceftazidime	>16, R
Ceftriaxone	>32, R
Ciprofloxacin	>2, R
Colistin	4, R
Ertapenem	≤0.25, S
Gentamicin	>8, R
Imipenem	≤0.25, S
Levofloxacin	>4, R
Meropenem	≤0.25, S
Nitrofurantoin	≤16, S
Piperacillin-tazobactam	4/4, S
Tetracycline	>8, R
Tobramycin	>8, R
Trimethoprim-sulfamethoxazole	>2/38, R

a MICs were determined using BD Phoenix (BD Diagnostics Systems, Hunt Valley, MD, USA) with panels NMIC/ID 133, except for colistin, for which determinations were performed using Etest and manual broth microdilution; both gave MICs of colistin of 4 μg/ml. R = resistant, I = intermediate, and S = susceptible, based on CLSI guidelines (except for colistin, where EUCAST breakpoints are used).

E. coli MRSN 388634 belonged to sequence type 457 (ST457), a rare E. coli ST first identified in 2008 from a urine culture in the United Kingdom (7). It was subsequently identified from a bloodstream culture in Italy, where it was found to harbor the carbapenemase genes *bla*_KPC-3_ and *bla*_CTX-M-55_ (8). MRSN 388634 carried 15 antibiotic resistance genes, which were harbored on two plasmids, but no carbapenemases (Table 2).

**TABLE 2:** Characteristics of plasmids in E. coli MRSN 388634

Plasmid name	Size (kb)	Inc^*a*^	Copy no.^*b*^	Antibiotic resistance genes^*c*^
pMR0516mcr	225.7	F18:A-:B1	2	*strA*, *strB*, *bla*_CTX-M-55_, *bla*_TEM-1B_, ***mcr-1***, *sul2*, *tet*(A), *dfrA14*
pMR0416ctx	47	N	1	*aac(3)-IVa*, *aph(4)-Ia*, *bla*_CTX-M-14_, *fosA3*, *mph*(A), *floR*, *sul2*

a Data represent plasmid incompatibility (Inc) group designations, as determined by Plasmid Finder version 1.2 (10).

b Data represent average numbers of copies per cell, normalized to the chromosomal read coverage.

c The gene of interest is indicated in bold.

**FIG 1:**
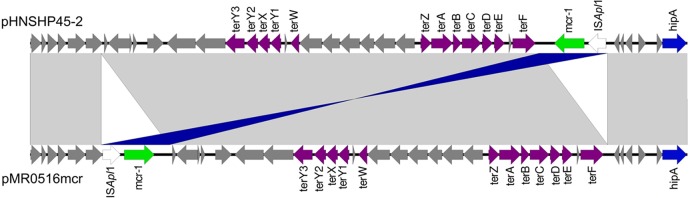
Comparison of the homologous regions containing *mcr-1* shared by pMR0516mcr and pHNSHP45-2. Open arrows represent coding sequences (green arrows, *mcr-1*; white arrows, IS*apl1*; purple arrows, metabolic function; blue arrows, plasmid replication and maintenance; gray arrows, hypothetical and unclassified) and indicate direction of transcription. The arrow size is proportional to the gene length. The gray and blue areas between pMR0516mcr and pHNSHP45-2 indicate nucleotide identity of >99.9% by BLASTN.

The first plasmid, pMR0516mcr, was 225,707 bp in size and belonged to incompatibility group F18:A-:B1 (9). BLAST analysis indicated that pMR0516mcr represented a novel IncF plasmid. Notably, it shares 89 kb of homologous sequence with pHNSHP45-2, a *mcr-1*-carrying IncHI2 plasmid described by Liu and colleagues (1). This shared sequence contains *mcr-1* in association with IS*Apl1* (1), but in pMR0516mcr it is in a different location and orientation (Fig. 1). pMR0516mcr also carried 7 additional antibiotic resistance genes, including the ESBL gene *bla*_CTX-M-55_ (Table 2). The second plasmid, pMR0416ctx, was ∼47 kb in size and was assigned to IncN (Table 2). It carried 7 antibiotic resistance genes, including *bla*_CTX-M-14_. A complete description of both plasmids is under preparation.

To the best of our knowledge, this is the first report of *mcr-1* in the United States. The epidemiology of MRSN 388634 is noteworthy; the isolate was submitted from a clinic in Pennsylvania, and the patient reported no travel history within the prior 5 months. To date, a further 20 ESBL-producing E. coli isolates from patients at the WRNMMC have tested negative for *mcr-1* and have been colistin sensitive. However, as testing has been ongoing for only 3 weeks, it remains unclear what the true prevalence of *mcr-1* is in the population. The association between *mcr-1* and IncF plasmids is concerning, as these plasmids are vehicles for the dissemination of antibiotic resistance and virulence genes among the Enterobacteriaceae (9). Continued surveillance to determine the true frequency for this gene in the United States is critical.

### Nucleotide sequence accession numbers.

The Short Read Archive (SRA) file for MRSN 388623 has been deposited at GenBank with accession number SRP075674. The complete sequence of pMR0516mcr has been deposited at GenBank with accession no. KX276657.
